# Quality appraisal of workers’ wellbeing measures: a systematic review protocol

**DOI:** 10.1186/s13643-018-0905-4

**Published:** 2018-12-20

**Authors:** Rebecca J. Jarden, Margaret Sandham, Richard J. Siegert, Jane Koziol-McLain

**Affiliations:** 1 University of Melbourne, Alan Gilbert Building, 161 Barry St, Carlton, Victoria 3053 Australia; 2 Auckland University of Technology (AUT), North Shore Campus, 90 Akoranga Drive, Northcote, Auckland, 0627 New Zealand

**Keywords:** Work wellbeing, Employee wellbeing, Measures, Systematic review, Quality appraisal

## Abstract

**Background:**

Measuring wellbeing has never been so important. With the rapid growth of workplace wellbeing interventions, determining their effectiveness is not only good science but also good practice. A wide variety of wellbeing measures exist in the literature but it is not always clear what they are measuring, nor which measures best meet study objectives. This study seeks to identify the most valid and reliable measure/s of workers’ wellbeing.

**Methods:**

Measures will be included if they were (1) designed for measuring workers’ wellbeing and (2) available in English. We will use a three-staged electronic search strategy to identify studies that include measures that meet the inclusion criteria: (1) electronic bibliographic databases for published work, (2) reference lists of studies with included measures, and (3) the reference list of previously published reviews. The following electronic bibliographic databases will be searched: OVID: psycINFO, psycTESTS, Cochrane library, AMED, Health and Psychosocial instruments; PubMed; PubPsych; Europe PMC; Scopus and Google Scholar. Database key search terms will include [*wellbeing OR* “*well-being*”] AND [employee* OR worker* OR staff OR personnel], and a validated search filter will be applied for the measurement properties. The methodological quality of the included studies will be assessed and rated. Then, this quality assessment of the included studies will be considered in the quality assessment of the measurement instruments. Finally, recommendations for the most appropriate instrument to measure workers’ wellbeing will be reported.

**Discussion:**

This systematic review will evaluate the quality of instruments that measure workers’ wellbeing. The findings of this review will improve clarity for researchers and clinicians in the appropriate instrument selection in the measurement of workers’ wellbeing.

**Systematic review registration:**

PROSPERO CRD42018079044

**Electronic supplementary material:**

The online version of this article (10.1186/s13643-018-0905-4) contains supplementary material, which is available to authorized users.

## Background

### Rationale

With the rapid growth of workplace wellbeing interventions, determining their effectiveness is not only good science but also good practice. Wellbeing research continues to evolve; thus, it is likely that there will be advances in measurement. With the growing list of wellbeing measures (e.g., see [1, 2]), identifying and selecting the most appropriate instruments for effectiveness evaluations in the workplace have never been so important. Whilst a wide variety of wellbeing measures exist in the literature, it is not always clear what they are measuring, nor which measures best meet study objectives. For example, a recent systematic review of longitudinal studies that investigated workers’ wellbeing constructs found the majority of the 40 identified studies focussed on illbeing, or the “negative side” of employee wellbeing (e.g., burnout [3]). This systematic review protocol, and subsequent systematic review, will provide a unique opportunity to provide future rigorous updates as the work wellbeing science grows. The study will improve clarity for researchers and clinicians in the appropriate instrument selection in the measurement of workers’ (or employees’) wellbeing.

The construct of workers’ wellbeing is described as rich and multifaceted, with key features scaffolding individual, team and organisational levels, inclusive of factors that transcend work (the role), workers (the individuals and teams), and workplaces (organisations) [4]. The factors associated with wellbeing differ for different occupational groups [5]. For professionals, the greatest amount of variance in job satisfaction is due to the five factors of work-life balance, satisfaction with education, being engaged, and experiencing meaning, purpose and autonomy [5]. For labourers, these factors were work-life balance, being absorbed, experiencing meaning and purpose, feeling respected, and having self-esteem [5]. For nurses, these factors included workplace characteristics, the ability to cope with changing demands and feedback loops [6]. The largely Western theoretical models and definitions of work wellbeing are also varied [7–9]. Key factors are thought to include subjective wellbeing, including job satisfaction, attitudes and affect; eudiamonic wellbeing including engagement, meaning, growth, intrinsic motivation and calling; and social wellbeing such as quality connections and satisfaction with co-workers [10]. Laine and Rinne [11] add to these factors in their ‘discursive’ definition which encompasses healthy living/working, work/family roles, leadership/management styles, human relations/social factors, work-related factors, working life uncertainties, and personality/individual factors. Work-related quality of life (WRQoL) adds further factors, including general wellbeing, home-work interface, job and career satisfaction, control at work, working conditions, and stress at work [12]. Given the breadth of these factors, and the disparity in theoretical models and definitions of what workers’ wellbeing is, selecting instruments for the measurement of workers’ wellbeing is challenging. The most appropriate instrument to measure the construct may require a selection of unidimensional (sub) scales, similar to the measurement of wellbeing [13] and WRQoL [12]. It is expected that two different instruments that are intended to measure the same construct of “workers’ wellbeing” should correlate. Thus, we will test the a priori hypothesis: instruments intending to assess the same construct of “workers’ wellbeing” will be strongly positively correlated. For this review, the aim is to evaluate the measurement properties of instruments that measure the broader construct of workers’ wellbeing (e.g., the Workplace Well-being Index [14, 15]). Any identifiable sub-scales within the instruments will be individually reported. Specifically, the objectives are to (1) systematically identify studies that measure workers’ wellbeing, (2) critically appraise the methodological quality of the studies, (3) critically appraise the workers’ wellbeing instrument properties and, (4) recommend the most appropriate instruments to measure workers’ wellbeing.

## Methods

This review protocol followed the Preferred Reporting Items for Systematic review and Meta-Analysis Protocols 2015 checklist (PRISMA-P; [16, 17]). Any protocol amendments will be documented in the systematic review.

### Review inclusion and exclusion criteria

#### Types of instruments

Eligible workers’ wellbeing data collection instruments include interviewer-administered, self-administered, or computer-administered. Examples include an online survey, a written questionnaire completed by a worker, or a worker’s responses to an interviewer’s questions.

#### Study design

Eligible studies will be those published as a full-text original article that (1) use a data collection instrument to investigate workers’ wellbeing (e.g., survey, interview or questionnaire) and (2) report measurement properties to enable reviewers to determine reliability (internal consistency, reliability, and measurement error/s), validity (content, criterion, and construct), responsiveness, and interpretability.

#### Settings and participants

This review will identify wellbeing outcome measures for workers’. Wellbeing measures may have been applied to any workplace (a workplace is defined as a place where a worker goes to carry out work [18]).

#### Types of wellbeing outcome measures

Instruments measuring workers’ wellbeing as an outcome will be eligible for inclusion. As a consequence of the disparate theoretical views and definitions of both wellbeing [19–21] and work wellbeing [7–9, 11, 22], for the purpose of this review, the terms used are very specific. For a measure to be included, the term ‘wellbeing’ must be specifically stated as either ‘wellbeing’, ‘well-being’ or ‘well being’. The term ‘workers’ must be specifically stated as either ‘employee*’, ‘worker*’, ‘staff’ or ‘personnel’.

Studies published in languages other than English will be excluded. Abstracts, books, theses and conference proceedings will be excluded.

### Information sources

The following electronic bibliographic databases will be searched: OVID: psycINFO, psycTESTS, Cochrane library, AMED, Health and psychosocial instruments; PubMed; PubPsych; Europe PMC; Scopus and Google Scholar. No date range will be applied.

### Search strategy

A three-staged search strategy will be used to identify studies that include measures meeting the inclusion criteria: (1) electronic bibliographic databases for published work, (2) reference lists of studies with included measures, and (3) the reference list of previously published reviews.

### Search terms

Database key search terms will include [*wellbeing OR* ‘*well-being*’] AND [employee* OR worker* OR staff OR personnel]. Search terms for measurement properties of measurement instruments will draw from the ‘precise search filter for measurement properties’ and ‘exclusion filter’ [23] (see sample search strategy, Additional file 1).

### Data management

References identified in the search strategy will be exported to EndNote X8 bibliographic software, and duplicates will be removed

### Selection process

Titles and abstracts will be screened by two independent reviewers. The full-text documents of these potentially relevant studies will then be independently screened against the eligibility criteria by two reviewers. Any disagreement will be resolved through consensus of the wider research team. Findings from the search will be presented in a Preferred Reporting Items for Systematic review and Meta-Analysis (PRISMA) flow chart [24].

### Data collection process

Data will be extracted into Microsoft Excel 2016 tables adopted from the COnsensus-based Standards for the selection of health status Measurement Instruments (COSMIN) methodology user guide [25]. Example tables which include variables to be extracted are presented in Additional file 1. The data tables will be checked for accuracy and completeness by a second reviewer.

### Assessment of the methodological quality of the included studies

The methodological quality of each single study on a measurement property will be assessed using the COSMIN risk of bias checklist for systematic reviews of Patient-Reported Outcome Measures (PROMs; [26]). For this review, in accordance with the COSMIN methodology recommendation, the term ‘Patient’ in ‘PROMs’ is considered synonymous with the population group for this study, ‘Worker’. The COSMIN checklist includes 10 boxes, two for content validity, three for internal structure, and five for the remaining measurement properties of reliability, measurement error, criterion validity, hypotheses testing for construct validity and responsiveness [26]. In terms of content validity, the COSMIN criteria and rating system for evaluating the content validity of PROMs will be used. Then two reviewers will independently rate each study’s quality for each measurement property using the COSMIN checklist 4-point rating scale as either very good, adequate, doubtful or inadequate [27]. A third reviewer will be consulted if any disagreement arises.

### Evaluation of the study results against criteria for good measurement properties

The quality of the measurement instruments will be rated as either sufficient, insufficient or indeterminate against the recently updated and published (see Table 2, p. 7; [28]) criteria of good measurement properties [29].

### Data synthesis

Data will be presented in summary of findings tables such as those presented in Additional file 1. The results from different studies on each measurement property will be quantitatively pooled in a meta-analysis, for example, by calculating weighted means and confidence intervals. If the data do not support meta-analysis, they will be summarised, for example, by providing ranges for interpretability values and percentages of confirmed hypotheses for construct validity.

### Confidence in cumulative evidence

The evidence will be summarised and the quality graded using four of the five factors in the Grading of Recommendations Assessment, Development, and Evaluation (GRADE; [30]) approach: risk of bias (i.e., methodological quality of the studies), inconsistency (i.e., unexplained inconsistency of results across studies), imprecision (i.e., total sample size of the available studies), and indirectness (i.e., evidence from different populations than the population of interest in the review) [27]. Results of all available studies on each measurement property will be quantitatively pooled or qualitatively summarised and compared against the criteria for good measurement properties to determine whether overall the measurement property of the measure is sufficient, insufficient, inconsistent, or indeterminate. If ratings per study are all sufficient (or all insufficient), results can be statistically pooled and the overall rating will be deemed sufficient (or insufficient) according to the criteria of good measurement properties. If results are inconsistent, this will be explored to determine explanations for inconsistency. Where explanations are determined, overall ratings will be provided for relevant subgroups with consistent results. Where no explanation is determined, the overall rating will be inconsistent. Where there is insufficient information available, the overall rating will be indeterminate. Overall ratings of each measurement property and the grading for the quality of the evidence will indicate reviewer confidence that pooled results and overall ratings are trustworthy. Where the overall rating for a specific measurement property is indeterminate, there will be no grading of the quality of the evidence as the quality of the measure cannot be judged. The quality assessment will be undertaken by two reviewers independently, and a third reviewer will be consulted in the event of unresolved disagreement.

## Discussion

This review systematically identifies and appraises measures that intend to assess the specific construct of workers’ wellbeing. The results will elucidate the existing specific measures of workers’ wellbeing and the quality of their measurement properties. This is an important first step to support future workers’ wellbeing researchers to identify and select the most appropriate instruments for effectiveness evaluations.

## Ethics

Ethical approval is not required for this systematic review that identifies, appraises, and synthesises published data.

## Dissemination

Findings from this review will be published in a peer-reviewed journal.

## Additional file

Additional file 1: Sample search strategy and sample data extraction, results and summary tables. (DOCX 42 kb)

## Abbreviations

COSMIN: COnsensus-based Standards for the selection of health status Measurement INstruments framework
GRADE: Grading of Recommendations Assessment, Development, and Evaluation
PRISMA: Preferred Reporting Items for Systematic review and Meta-Analysis
PRISMA-P: Preferred Reporting Items for Systematic review and Meta-Analysis Protocols
PROMs: Patient-Reported Outcome Measures
WRQoL: Work-related quality of life

## References

[1] Carlson J, Geisinger K, Jonson J (2017). Twentieth Mental Measurements Yearbook.

[2] Linton M, Dieppe P, Medina-Lara A, Watson L, Crathorne L (2016). Review of 99 self-report measures for assessing well-being in adults: exploring dimensions of well-being and developments over time. BMJ Open.

[3] Mäkikangas A, Kinnunen U, Feldt T, Schaufeli W (2016). The longitudinal development of employee well-being: a systematic review. Work Stress.

[4] Jarden R, Sandham M, Siegert R, Koziol-McLain J (2018). Intensive care nurse conceptions of wellbeing: a prototype analysis. Nurs Crit Care.

[5] Hamling K, Jarden A, Schofield G (2015). Recipes for occupational wellbeing: an investigation of the associations with wellbeing in New Zealand workers. N Z J Hum Resour Manage.

[6] Brand S, Fleming L, Wyatt K (2015). Tailoring healthy workplace interventions to local healthcare settings: a complexity theory-informed workplace of well-being framework. Sci World J.

[7] Grant A, Christianson M, Price R (2007). Happiness, health, or relationships? Managerial practices and employee well-being tradeoffs. Acad Manag Perspect.

[8] Dewe P, Kompier M (2008). Foresight mental capital and wellbeing project. Wellbeing at work: future challenges.

[9] Page K, Vella-Brodrick D (2009). The “what,” “why” and “how” of employee wellbeing: a new model. Soc Indic Res.

[10] Lawrie E, Tuckey M, Dollard M. Job design for mindful work: the boosting effect of psychosocial safety climate. 2018;23:483–95. 10.1037/ocp0000102 29283600

[11] Laine P, Rinne R (2015). Developing wellbeing at work: emerging dilemmas. Int J Wellbeing.

[12] Easton S, Van Laar D (2018). User manual for the work-related quality of life (WRQoL) scale: a measure of quality of working life. 2018 edition.

[13] Jarden A, Jarden R, Oades L, Steger M, Della-Fave A, Passmore J (2016). Positive psychological assessment for the workplace. The Wiley Blackwell Handbook of the psychology of positivity and strengths-based approaches at work.

[14] Page K. Subjective wellbeing in the workplace. Unpublished honours thesis. Melbourne: Deakin University; 2005.

[15] Page K, Vella-Brodrick D (2013). The working for wellness program: RCT of an employee well-being intervention. J Happiness Stud.

[16] Moher D, Shamseer L, Clarke M, Ghersi D, Liberati A, Petticrew M, Shekelle P, Stewart L (2015). Preferred reporting items for systematic review and meta-analysis protocols (PRISMA-P) 2015 statement. Syst Rev.

[17] Shamseer L, Moher D, Clarke M, Ghersi D, Liberati A, Petticrew M, Shekelle P, Stewart L (2015). Preferred reporting items for systematic review and meta-analysis protocols (PRISMA-P) 2015: elaboration and explanation. BMJ.

[18] Health and Safety at Work Act 2015. No. 70. New Zealand: New Zealand Parliamentary Counsel Office; 2015.

[19] Dodge R, Daly A, Huyton J, Sanders L (2012). The challenge of defining wellbeing. Int J Wellbeing.

[20] Huppert F (2009). Psychological well-being: evidence regarding its causes and consequences. Appl Psychol Health Well-Being.

[21] Hone L, Schofield G, Jarden A (2015). Conceptualizations of wellbeing: insights from a prototype analysis on New Zealand workers. N Z J Hum Resour Manage.

[22] Fisher C, Cooper CL (2014). Conceptualizing and measuring wellbeing at work. Wellbeing: a complete reference guide.

[23] Terwee C, Jansma E, Riphagen I, de Vet H (2009). Development of a methodological PubMed search filter for finding studies on measurement properties of measurement instruments. Qual Life Res.

[24] Moher D, Liberati A, Tetzlaff J, Altman DG, Altman D, Antes G, Atkins D, Barbour V, Barrowman N, Berlin JA, et al. Preferred reporting items for systematic reviews and meta-analyses: the PRISMA statement. PLoS Med. 2009;6:1-6.

[25] Mokkink L, Prinsen C, Patrick D, Alonso J, Bouter L, de Vet H, Terwee C (2018). COSMIN methodology for systematic reviews of Patient-Reported Outcome Measures (PROMs): user manual.

[26] Mokkink L, de Vet H, Prinsen C, Patrick D, Alonso J, Bouter L, Terwee C (2018). COSMIN risk of bias checklist for systematic reviews of patient-reported outcome measures. Qual Life Res.

[27] Prinsen C, Mokkink L, Bouter L, Alonso J, Patrick D, de Vet H, Terwee C (2018). COSMIN guideline for systematic reviews of patient-reported outcome measures. Qual Life Res.

[28] Prinsen C, Vohra S, Rose M, Boers M, Tugwell P, Clarke M, Williamson P, Terwee C. How to select outcome measurement instruments for outcomes included in a “Core Outcome Set” - a practical guideline. BMC Trials. 2016;17:449–59. 10.1186/s13063-016-1555-2 PMC5020549 27618914

[29] Terwee C, Bot S, de Boer M, van der Windt D, Knol D, Dekker J, Bouter L, de Vet H (2007). Quality criteria were proposed for measurement properties of health status questionnaires. J Clin Epidemiol.

[30] Guyatt G, Oxman A, Akl E, Kunz R, Vist G, Brozek J, Norris S, Falck-Ytter Y, Glasziou P, deBeer H (2011). GRADE guidelines: 1. Introduction—GRADE evidence profiles and summary of findings tables. J Clin Epidemiol.

