# Supplementary material for: Quality appraisal of workers’ wellbeing measures: a systematic review protocol
Source: Syst Rev. 2018 Dec 20;7:240. doi: 10.1186/s13643-018-0905-4 (PMC6300880; doi:10.1186/s13643-018-0905-4)
Supplement: Supplementary file 1 — Sample search strategy and sample data extraction, results and summary tables. (DOCX 42 kb) [file 13643_2018_905_MOESM1_ESM.docx]

Appendix A. Sample search strategy for PubMed^TM^ workers’ wellbeing measures systematic review

| Search term number | Search terms |
| --- | --- |
| 1 | wellbeing OR well-being |
| 2 | employee* OR worker* OR staff OR personnel |
| 3 | instrumentation[sh] OR Validation Studies[pt] OR ‘‘reproducibility of results’’[MeSH Terms] OR reproducib*[ tiab] OR ‘‘psychometrics’’[MeSH] OR psychometr*[ tiab] OR clinimetr*[tiab] OR clinometr*[tiab] OR ‘‘observer variation’’[MeSH] OR observer variation[tiab] OR ‘‘discriminant analysis’’[MeSH] OR reliab*[tiab] OR valid*[tiab] OR coefficient[tiab] OR ‘‘internal consistency’’[ tiab] OR (cronbach*[tiab] AND (alpha[tiab] OR alphas[tiab])) OR ‘‘item correlation’’[tiab] OR ‘‘item correlations’’[ tiab] OR ‘‘item selection’’[tiab] OR ‘‘item selections’’[ tiab] OR ‘‘item reduction’’[tiab] OR ‘‘item reductions’’[tiab] OR agreement[tw] OR precision[tw] OR imprecision[tw] OR ‘‘precise values’’[tw] OR test–retest [tiab] OR (test[tiab] AND retest[tiab]) OR (reliab*[tiab] AND (test[tiab] OR retest[tiab])) OR stability[tiab] OR interrater[tiab] OR inter-rater[tiab] OR intrarater[tiab] OR intra-rater[tiab] OR intertester[tiab] OR inter-tester[tiab] OR intratester[tiab] OR intra-tester[tiab] OR interobserver[ tiab] OR inter-observer[tiab] OR intraobserver[tiab] OR intra-observer[tiab] OR intertechnician[tiab] OR intertechnician[ tiab] OR intratechnician[tiab] OR intra-technician[ tiab] OR interexaminer[tiab] OR inter-examiner[tiab] OR intraexaminer[tiab] OR intra-examiner[tiab] OR interassay[ tiab] OR inter-assay[tiab] OR intraassay[tiab] OR intra-assay[tiab] OR interindividual[tiab] OR inter-individual[ tiab] OR intraindividual[tiab] OR intra-individual[tiab] OR interparticipant[tiab] OR inter-participant[tiab] OR intraparticipant[tiab] OR intra-participant[tiab] OR kappa[- tiab] OR kappa’s[tiab] OR kappas[tiab] OR ‘‘coefficient of variation’’[tiab] OR repeatab*[tw] OR ((replicab*[tw] OR repeated[tw]) AND (measure[tw] OR measures[tw] OR findings[tw] OR result[tw] OR results[tw] OR test[tw] OR tests[tw])) OR generaliza*[tiab] OR generalisa*[tiab] OR concordance[tiab] OR (intraclass[tiab] AND correlation*[ tiab]) OR discriminative[tiab] OR ‘‘known group’’ [tiab] OR ‘‘factor analysis’’[tiab] OR ‘‘factor analyses’’[tiab] OR ‘‘factor structure’’[tiab] OR ‘‘factor structures’’[tiab] OR dimensionality[tiab] OR subscale*[tiab] OR ‘‘multitrait scaling analysis’’[tiab] OR ‘‘multitrait scaling analyses’’[ tiab] OR ‘‘item discriminant’’[tiab]OR ‘‘interscale correlation’’[tiab] OR ‘‘interscale correlations’’[tiab] OR ((error[tiab] OR errors[tiab]) AND (measure*[tiab] OR correlat*[tiab] OR evaluat*[tiab] OR accuracy[tiab] OR accurate[tiab] OR precision[tiab] OR mean[tiab])) OR ‘‘individual variability’’[tiab] OR ‘‘interval variability’’[ tiab] OR ‘‘rate variability’’[tiab] OR ‘‘variability analysis’’[ tiab] OR (uncertainty[tiab] AND (measurement[tiab] 1122 Qual Life Res (2009) 18:1115–1123 123 OR measuring[tiab])) OR ‘‘standard error of measurement’’[ tiab] OR sensitiv*[tiab] OR responsive*[tiab] OR (limit[tiab] AND detection[tiab]) OR ‘‘minimal detectable concentration’’[tiab]ORinterpretab*[tiab] OR (small*[tiab] AND (real[tiab] OR detectable[tiab]) AND (change[tiab] OR difference[tiab])) OR ‘‘meaningful change’’[tiab] OR ‘‘minimal important change’’[tiab] OR ‘‘minimal important difference’’[tiab] OR ‘‘minimally important change’’[tiab] OR ‘‘minimally important difference’’[tiab] OR ‘‘minimal detectable change’’[tiab] OR ‘‘minimal detectable difference’’[ tiab] OR ‘‘minimally detectable change’’[tiab] OR ‘‘minimally detectable difference’’[tiab] OR ‘‘minimal real change’’[tiab] OR ‘‘minimal real difference’’[tiab] OR ‘‘minimally real change’’[tiab] OR ‘‘minimally real difference’’[ tiab] OR ‘‘ceiling effect’’[tiab] OR ‘‘floor effect’’ [tiab] OR ‘‘Item response model’’[tiab] OR IRT[tiab] OR Rasch[tiab] OR ‘‘Differential item functioning’’[tiab] OR DIF[tiab] OR ‘‘computer adaptive testing’’[tiab] OR ‘‘item bank’’[tiab] OR ‘‘cross-cultural equivalence’’[tiab] |
| 4 | NOT ‘‘addresses’’[Publication Type] OR ‘‘biography’’[Publication Type] OR ‘‘case reports’’[Publication Type] OR ‘‘comment’’[Publication Type] OR ‘‘directory’’[Publication Type] OR ‘‘editorial’’[Publication Type] OR ‘‘festschrift’’[ Publication Type] OR ‘‘interview’’[Publication Type] OR ‘‘lectures’’[Publication Type] OR ‘‘legal cases’’[Publication Type] OR ‘‘legislation’’[Publication Type] OR ‘‘letter’’[Publication Type] OR ‘‘news’’[Publication Type] OR ‘‘newspaper article’’[Publication Type] OR ‘‘patient education handout’’[Publication Type] OR ‘‘popular works’’[Publication Type] OR ‘‘congresses’’ [Publication Type] OR ‘‘consensus development conference’’[ Publication Type] OR ‘‘consensus development conference, nih’’[Publication Type] OR ‘‘practice guideline’’[ Publication Type]) NOT (‘‘animals’’[MeSH Terms] NOT ‘‘humans’’[MeSH Terms] |
| 5 | *1 AND 2 AND 3 AND 4* |

**Example:**

(((((instrumentation[sh] OR Validation Studies[pt] OR ‘‘reproducibility of results’’[MeSH Terms] OR reproducib*[ tiab] OR ‘‘psychometrics’’[MeSH] OR psychometr*[ tiab] OR clinimetr*[tiab] OR clinometr*[tiab] OR ‘‘observer variation’’[MeSH] OR observer variation[tiab] OR ‘‘discriminant analysis’’[MeSH] OR reliab*[tiab] OR valid*[tiab] OR coefficient[tiab] OR ‘‘internal consistency’’[ tiab] OR (cronbach*[tiab] AND (alpha[tiab] OR alphas[tiab])) OR ‘‘item correlation’’[tiab] OR ‘‘item correlations’’[ tiab] OR ‘‘item selection’’[tiab] OR ‘‘item selections’’[ tiab] OR ‘‘item reduction’’[tiab] OR ‘‘item reductions’’[tiab] OR agreement[tw] OR precision[tw] OR imprecision[tw] OR ‘‘precise values’’[tw] OR test–retest [tiab] OR (test[tiab] AND retest[tiab]) OR (reliab*[tiab] AND (test[tiab] OR retest[tiab])) OR stability[tiab] OR interrater[tiab] OR inter-rater[tiab] OR intrarater[tiab] OR intra-rater[tiab] OR intertester[tiab] OR inter-tester[tiab] OR intratester[tiab] OR intra-tester[tiab] OR interobserver[ tiab] OR inter-observer[tiab] OR intraobserver[tiab] OR intra-observer[tiab] OR intertechnician[tiab] OR intertechnician[ tiab] OR intratechnician[tiab] OR intra-technician[ tiab] OR interexaminer[tiab] OR inter-examiner[tiab] OR intraexaminer[tiab] OR intra-examiner[tiab] OR interassay[ tiab] OR inter-assay[tiab] OR intraassay[tiab] OR intra-assay[tiab] OR interindividual[tiab] OR inter-individual[ tiab] OR intraindividual[tiab] OR intra-individual[tiab] OR interparticipant[tiab] OR inter-participant[tiab] OR intraparticipant[tiab] OR intra-participant[tiab] OR kappa[- tiab] OR kappa’s[tiab] OR kappas[tiab] OR ‘‘coefficient of variation’’[tiab] OR repeatab*[tw] OR ((replicab*[tw] OR repeated[tw]) AND (measure[tw] OR measures[tw] OR findings[tw] OR result[tw] OR results[tw] OR test[tw] OR tests[tw])) OR generaliza*[tiab] OR generalisa*[tiab] OR concordance[tiab] OR (intraclass[tiab] AND correlation*[ tiab]) OR discriminative[tiab] OR ‘‘known group’’ [tiab] OR ‘‘factor analysis’’[tiab] OR ‘‘factor analyses’’[tiab] OR ‘‘factor structure’’[tiab] OR ‘‘factor structures’’[tiab] OR dimensionality[tiab] OR subscale*[tiab] OR ‘‘multitrait scaling analysis’’[tiab] OR ‘‘multitrait scaling analyses’’[ tiab] OR ‘‘item discriminant’’[tiab]OR ‘‘interscale correlation’’[tiab] OR ‘‘interscale correlations’’[tiab] OR ((error[tiab] OR errors[tiab]) AND (measure*[tiab] OR correlat*[tiab] OR evaluat*[tiab] OR accuracy[tiab] OR accurate[tiab] OR precision[tiab] OR mean[tiab])) OR ‘‘individual variability’’[tiab] OR ‘‘interval variability’’[ tiab] OR ‘‘rate variability’’[tiab] OR ‘‘variability analysis’’[ tiab] OR (uncertainty[tiab] AND (measurement[tiab] 1122 Qual Life Res (2009) 18:1115–1123 123 OR measuring[tiab])) OR ‘‘standard error of measurement’’[ tiab] OR sensitiv*[tiab] OR responsive*[tiab] OR (limit[tiab] AND detection[tiab]) OR ‘‘minimal detectable concentration’’[tiab]ORinterpretab*[tiab] OR (small*[tiab] AND (real[tiab] OR detectable[tiab]) AND (change[tiab] OR difference[tiab])) OR ‘‘meaningful change’’[tiab] OR ‘‘minimal important change’’[tiab] OR ‘‘minimal important difference’’[tiab] OR ‘‘minimally important change’’[tiab] OR ‘‘minimally important difference’’[tiab] OR ‘‘minimal detectable change’’[tiab] OR ‘‘minimal detectable difference’’[ tiab] OR ‘‘minimally detectable change’’[tiab] OR ‘‘minimally detectable difference’’[tiab] OR ‘‘minimal real change’’[tiab] OR ‘‘minimal real difference’’[tiab] OR ‘‘minimally real change’’[tiab] OR ‘‘minimally real difference’’[ tiab] OR ‘‘ceiling effect’’[tiab] OR ‘‘floor effect’’ [tiab] OR ‘‘Item response model’’[tiab] OR IRT[tiab] OR Rasch[tiab] OR ‘‘Differential item functioning’’[tiab] OR DIF[tiab] OR ‘‘computer adaptive testing’’[tiab] OR ‘‘item bank’’[tiab] OR ‘‘cross-cultural equivalence’’[tiab]))) AND ((employee* OR worker* OR staff OR personnel))) AND (((wellbeing OR well-being)))) NOT ((‘‘addresses’’[Publication Type] OR ‘‘biography’’[Publication Type] OR ‘‘case reports’’[Publication Type] OR ‘‘comment’’[Publication Type] OR ‘‘directory’’[Publication Type] OR ‘‘editorial’’[Publication Type] OR ‘‘festschrift’’[ Publication Type] OR ‘‘interview’’[Publication Type] OR ‘‘lectures’’[Publication Type] OR ‘‘legal cases’’[Publication Type] OR ‘‘legislation’’[Publication Type] OR ‘‘letter’’[Publication Type] OR ‘‘news’’[Publication Type] OR ‘‘newspaper article’’[Publication Type] OR ‘‘patient education handout’’[Publication Type] OR ‘‘popular works’’[Publication Type] OR ‘‘congresses’’ [Publication Type] OR ‘‘consensus development conference’’[ Publication Type] OR ‘‘consensus development conference, nih’’[Publication Type] OR ‘‘practice guideline’’[ Publication Type]) NOT (‘‘animals’’[MeSH Terms] NOT ‘‘humans’’[MeSH Terms]))

Appendix B. Example data extraction, results and summary tables.

Example table of characteristics of the included PROMs adapted from COSMIN manual Appendix 3 [25]

| PROM (reference to first article) | Construct(s) | Target population | Mode of administration | Recall period | (Sub)scale(s) (number of items) | Response options | Range of scores/scoring | Original language | Available translations |
| --- | --- | --- | --- | --- | --- | --- | --- | --- | --- |
|  |  |  |  |  |  |  |  |  |  |
|  |  |  |  |  |  |  |  |  |  |

Example table of characteristics of the included study populations adapted from COSMIN manual Appendix 4 [25]

|  |  | Population | | | Instrument administration | | | |
| --- | --- | --- | --- | --- | --- | --- | --- | --- |
| PROM | Ref | N | Age; Mean (SD,range) year | Gender % female | Setting | Country | Language | Response rate |
| A | 1 |  |  |  |  |  |  |  |

Example table of information to extract on interpretability of PROMs adapted from COSMIN manual Appendix 5 [25]

| PROM (ref) | Distribution of scores in the study population | Percentage of missing items and percentage of missing total scores | Floor and ceiling effects | Scores and change scores available for relevant (sub)groups | Minimal important change or minimal important difference | Information on response shift |
| --- | --- | --- | --- | --- | --- | --- |
| PROMA (ref 1) |  |  |  |  |  |  |

Example table of information to extract on feasibility of PROMs adapted from COSMIN manual Appendix 6 [25]

| Feasibility aspects | PROM A | PROM B | PROM C | PROM D |
| --- | --- | --- | --- | --- |
| Worker’s comprehensibility |  |  |  |  |
| Type and ease of administration |  |  |  |  |
| Completion time |  |  |  |  |
| Ease of standardisation |  |  |  |  |
| Ease of score calculation |  |  |  |  |
| Copyright |  |  |  |  |
| Cost of an instrument |  |  |  |  |
| Required equipment |  |  |  |  |
| Availability in different settings |  |  |  |  |

Example table of results of studies on measurement properties adapted from COSMIN manual Appendix 7 [25]

| PROM | Country (language) in which evaluated | Structural validity | | | Internal consistency | | | Cross-cultural validity/measurement invariance | | | Reliability | | |
| --- | --- | --- | --- | --- | --- | --- | --- | --- | --- | --- | --- | --- | --- |
|  |  | n | Meth quality | Result (rating) | n | Meth quality | Result (rating) | n | Meth quality | Result (rating) | n | Meth quality | Result (rating) |
| … |  |  |  |  |  |  |  |  |  |  |  |  |  |
| … |  |  |  |  |  |  |  |  |  |  |  |  |  |
| Pooled or summary result (overall rating) | |  |  |  |  |  |  |  |  |  |  |  |  |

| PROM | Country (language) in which evaluated | Measurement error | | | Criterion validity | | | Hypotheses testing | | | Responsiveness | | |
| --- | --- | --- | --- | --- | --- | --- | --- | --- | --- | --- | --- | --- | --- |
|  |  | n | Meth quality | Result (rating) | n | Meth quality | Result (rating) | n | Meth quality | Result (rating) | n | Meth quality | Result (rating) |
| … |  |  |  |  |  |  |  |  |  |  |  |  |  |
| … |  |  |  |  |  |  |  |  |  |  |  |  |  |
| Pooled or summary result (overall rating) | |  |  |  |  |  |  |  |  |  |  |  |  |

Example summary of findings tables adapted from COSMIN manual Appendix 8 [25]

| Structural validity | Summary or pooled result | Overall rating | Quality of evidence |
| --- | --- | --- | --- |
| PROM A |  |  |  |
| PROM B |  |  |  |

| Internal consistency | Summary or pooled result | Overall rating | Quality of evidence |
| --- | --- | --- | --- |
| PROM A |  |  |  |
| PROM B |  |  |  |

| Cross-cultural validity/measurement invariance | Summary or pooled result | Overall rating | Quality of evidence |
| --- | --- | --- | --- |
| PROM A |  |  |  |
| PROM B |  |  |  |

| Reliability | Summary or pooled result | Overall rating | Quality of evidence |
| --- | --- | --- | --- |
| PROM A |  |  |  |
| PROM B |  |  |  |

| Measurement error | Summary or pooled result | Overall rating | Quality of evidence |
| --- | --- | --- | --- |
| PROM A |  |  |  |
| PROM B |  |  |  |

| Hypotheses testing | Summary or pooled result | Overall rating | Quality of evidence |
| --- | --- | --- | --- |
| PROM A |  |  |  |
| PROM B |  |  |  |

| Responsiveness | Summary or pooled result | Overall rating | Quality of evidence |
| --- | --- | --- | --- |
| PROM A |  |  |  |
| PROM B |  |  |  |
